# Association of the I1307K APC mutation with hereditary and sporadic breast/ovarian cancer: more questions than answers

**DOI:** 10.1054/bjoc.2000.1248

**Published:** 2000-06-15

**Authors:** R Gershoni-Baruch, Y Patael, Dagan, A Figer, L Kasinetz, E Kadouri, R Bruchim Bar Sade, E Friedman

**Affiliations:** Department of Genetics, Rambam Medical Center, Haifa, Israel; Bruce Rappoport Faculty of Medicine, Technion-Israel Institute of Technology, Haifa, Israel; Susanne Levy Gertner Oncogenetics Unit, Institute of Oncology, Rabin Medical Center, Petah Tikvah, Israel

**Keywords:** breast cancer, ovarian cancer, BRCA1, BRCA2, APC, I1307K polymorphism

## Abstract

The frequency of the APC I1307K mutation and its association with disease pattern was examined in 996 Ashkenazi women consisting of individuals with either sporadic (*n* = 382) or hereditary (*n* = 143) breast and/or ovarian cancer; asymptomatic BRCA1/2 mutation carriers (185delAG, 5382insC and 6174delT) (*n* = 53) and healthy controls (*n* = 418). The I1307K allele was equally distributed among women with sporadic (17/382; 4.6%) and inherited (10/143; 7%) breast and/or ovarian cancer irrespective of their being diagnosed before or after 42 years of age and among asymptomatic (7/53; 13.2%) and cancer manifesting BRCA1/2 carriers (10/143; 7%). Taken together, the prevalence of the I1307K allele was significantly higher in BRCA1/2 carriers compared to non-BRCA1/2 carriers (17/196; 8.7% and 40/800, 5%; respectively). The high prevalence of the I1307K allele among BRCA1/2 carriers is not associated with increased cancer risk but seems to be genetically connected because of Jewish ancestry. © 2000 Cancer Research Campaign


British Journal of Cancer (2000) 83(2), 153–155
© 2000 Cancer Research Campaign
doi: 10.1054/ bjoc.2000.1248, available online at http://www.idealibrary.com on IDEAL®

### Association of the I1307K APC mutation with hereditary and sporadic breast/ovarian cancer: more questions than answers

**R Gershoni-Baruch,[1,2] Y Patael,[3] Dagan,[1,2] A Figer,[4] L Kasinetz,[1] E Kadouri,[1] R Bruchim Bar Sade[3] and E Friedman[3]**

[1]Department of Genetics, Rambam Medical Center, Haifa, Israel; [2]Bruce Rappoport Faculty of Medicine, Technion-Israel Institute of Technology, Haifa, Israel; [3]Susanne Levy Gertner Oncogenetics Unit; [4]Institute of Oncology, Rabin Medical Center, Petah Tikvah, Israel

**Summary** The frequency of the APC I1307K mutation and its association with disease pattern was examined in 996 Ashkenazi women consisting of individuals with either sporadic ($n = 382$) or hereditary ($n = 143$) breast and/or ovarian cancer; asymptomatic BRCA1/2 mutation carriers (185delAG, 5382insC and 6174delT) ($n = 53$) and healthy controls ($n = 418$). The I1307K allele was equally distributed among women with sporadic (17/382; 4.6%) and inherited (10/143; 7%) breast and/or ovarian cancer irrespective of their being diagnosed before or after 42 years of age and among asymptomatic (7/53; 13.2%) and cancer manifesting BRCA1/2 carriers (10/143; 7%). Taken together, the prevalence of the I1307K allele was significantly higher in BRCA1/2 carriers compared to non-BRCA1/2 carriers (17/196; 8.7% and 40/800, 5%; respectively). The high prevalence of the I1307K allele among BRCA1/2 carriers is not associated with increased cancer risk but seems to be genetically connected because of Jewish ancestry. © 2000 Cancer Research Campaign

**Keywords**: breast cancer; ovarian cancer; BRCA1; BRCA2; APC; I1307K polymorphism

Females with BRCA1/2 mutations have a 40% to 60% lifetime risk of developing breast cancer and a 16% to 40% risk of developing ovarian cancer (Friedman et al, 1995; Levy-Lahad et al, 1997). The incomplete penetrance of the BRCA1/2 gene mutations suggests that other factors, genetic and non-genetic, determine the phenotypic expression of mutant BRCA1/2 alleles. Candidate 'modifier genes' to consider include genes with a known relevance to breast tumorigenesis (e.g., p53), genes which physically interact with BRCA1 or BRCA2 (e.g., RAD51), or those that have an in-vitro effect on the proliferation rate of breast epithelial cells (e.g., estrogen receptor, vitamin D receptor).

In Ashkenazi Jews three predominant founder mutations were described (185delAG and 5382insC in BRCA1 and 6174delT in BRCA2) (Friedman et al, 1995; Modan et al, 1996; Neuhausen et al, 1996; Tonin et al, 1996). In this population, one could consider as putative modifier genes, genes that display a high carrier state and are known to be associated with an increased risk for cancer. The recently described I1307K missense mutation in the APC gene, seems to fit into this category. This missense mutation has been detected in 28% of Jewish Ashkenazi individuals with familial colorectal cancer, and in 6–7% of the general Jewish Ashkenazi population (Laken et al, 1997; Woodage et al, 1998). The apparent mechanism underlying cancer predisposition is the creation of a homopolymer tract (A8), resulting in tissue-specific genomic instability, prone to acquiring somatic mutation (Laken et al, 1997). Several lines of evidence suggest that the APC gene might participate in breast cancer tumorigenesis. Loss of heterozygosity involving the APC gene has been reported in sporadic breast tumours (Thompson et al, 1993; Kashiwaba et al, 1994). Woodage and coworkers (1998) reported that I1307K mutation carriers are more likely to have a first degree relative with breast cancer. Redston and coworkers (1998) have affirmed that cancer risk conferred by mutant BRCA1/2 alleles is increased by the presence of the I1307K APC polymorphism.

To better delineate an association between the I1307K mutation and breast/ovarian cancer we determined the prevalence of this mutation in Jewish patients with inherited and sporadic breast and/or ovarian cancer.

*Received 2 June 1999*
*Revised 25 February 2000*
*Accepted 23 March 2000*

*Correspondence to:* E Friedman

### MATERIAL AND METHODS

### Patients and controls

**Patients** – 366 self-referred or physician-referred patients with either breast and/or ovarian cancer visiting the Oncogenetics services at Sheba and Rambam Medical Centers and 159 unselected patients with breast cancer seen at Sheba and Rambam Oncology Clinic during 1997 and 1998. All patients were systematically screened for the three predominant BRCA1 and BRCA2 mutations and for the I1307K APC mutation.

**Asymptomatic – carriers** 53 family members of mutation carriers or individuals referred because of a family history of breast/ovarian cancer.

**Controls** – 418 healthy Ashkenazi individuals who presented for genetic testing of common recessive diseases. These were systematically screened for the I1307K APC mutation only.

Data including demographics, histopathological information, treatment and outcome variables were collected and entered into a computerized database. All participants signed an informed consent form approved by the Institutional Review Board (IRB) and were genotyped for the three predominant founder mutations

**Table 1** Distribution of the I1307K allele among patients and healthy participants

| Groups | Subgroups | I1307K carriers | Total | % | 95% CI |
|---|---|---|---|---|---|
| Total | | 57 | 996 | 5.7 | 4.40–7.40 |
| Healthy controls | | 23 | 418 | 5.5 | 3.60–8.26 |
| Sporadic cases | | | | | |
| | Late onset breast cancer[a] | 15 | 276 | 5.4 | 3.18–8.99 |
| | Early onset breast cancer | 1 | 81 | 1.2 | 0.06–7.64 |
| | Ovarian cancer | 1 | 25 | 4 | 0.21–22.32 |
| | All | 17 | 382 | 4.6 | 2.70–7.17 |
| BRCA1/2 carriers | | | | | |
| | Late onset breast cancer[b] | 5 | 59 | 8.5 | 3.16–19.42 |
| | Early onset breast cancer[c] | 4 | 55 | 7.3 | 2.36–18.43 |
| | OC | 1 | 29 | 2.8 | 0.18–19.63 |
| | Asymptomatic | 7 | 53 | 13.2 | 5.91–25.95 |
| | All | 17 | 196 | 8.7 | 5.29–13.75 |

[a]Includes 2 patients with both breast and ovarian cancer. [b]Includes 10 patients with both breast and ovarian cancer. [c]Includes 4 patients with both breast and ovarian cancer.

**Table 2** Prevalence of the I1307K allele among breast and/or ovarian cancer patients and healthy controls with/without BRCA1/2 mutations.

| Groups | Total | BRCA1/2 carriers | | | | | Non-BRCA1/2 carriers | | | | | | | | |
|---|---|---|---|---|---|---|---|---|---|---|---|---|---|---|---|
| | | Total | I1307K carriers | | I1307K non-carriers | | Total | I1307K carriers | | I1307K non-carriers | | | | | |
| | | | No. | % | No. | % | | No. | % | No. | % | $\chi^2$ | p | OR | 95% CI |
| All patients | 525 | 143 | 10 | 7 | 133 | 93 | 382 | 17 | 4.4 | 365 | 95.6 | 1.07 | 0.2 | 1.5 | 0.7–3.4 |
| Breast cancer patients | 471 | 114 | 9 | 7.9 | 105 | 92.1 | 357 | 16 | 4.5 | 341 | 95.5 | 2 | 0.122 | 1.8 | 0.8–4.2 |
| Early onset breast cancer | 136 | 55 | 4 | 7.3 | 51 | 92.7 | 81 | 1 | 1.2 | 80 | 98.8 | 3.4 | 0.086 | 6.275 | 0.7–57.7 |
| Late onset breast cancer | 335 | 59 | 5 | 8.5 | 54 | 92.5 | 276 | 15 | 5.4 | 261 | 94.6 | 0.8 | 0.265 | 1.6 | 0.6–4.6 |
| Ovarian cancer patients | 54 | 29 | 1 | 2.8 | 28 | 98.2 | 25 | 1 | 4 | 24 | 96 | 0.01 | 0.72 | 0.86 | 0.02–33.5 |
| Healthy participants | 471 | 53 | 7 | 13.2 | 46 | 76.8 | 418 | 23 | 5.5 | 395 | 94.5 | 4.68 | 0.04 | 2.6 | 1.1–6.4 |
| Total | 996 | 196 | 17 | 8.7 | 179 | 91.3 | 800 | 40 | 5 | 760 | 95 | 3.615 | 0.046 | 1.7 | 1–3.1 |

in BRCA1 (185delAG and 5382insC) and BRCA2 (6174delT) and for I1307K APC mutation.

### Genotyping

All genotyping was performed on DNA extracted from lymphocytes.

The 185delAG, 5382insC (BRCA1) and 6174delT (BRCA2) were detected by PCR amplification with specific primers that produce a modified restriction enzyme digest made to distinguish the wild-type allele from the mutant allele, as previously described (Abeliovich et al, 1997; Rohlfs et al, 1997). The I1307K mutations (APC) was detected using specific primers that produce a modified restriction enzyme digest made to distinguish the wild-type allele from the mutant allele (unpublished data). Alternatively, the APC I1307K mutation was detected by DGGE and direct sequencing as previously described (Patael et al, 1999).

### Statistical analysis

The $\chi^2$ (Pearson and Fisher exact) tests were used for comparisons between groups. Proportions and 95% confidence intervals were calculated. Odd ratios and 95% confidence intervals were used to evaluate the prevalence of the APC I1307K mutation in BRCA1/2 carriers and non-carriers whether patients or controls.

### RESULTS

### Characteristics (tables 1.2)

**Patients** – 525 patients (mean age 50.9 ± 12.27; range 26–86): 471 with breast cancer (including 16 with both breast and ovarian cancer); of whom 136 were diagnosed prior to age 42 years and 335 diagnosed after that age; and 54 with ovarian cancer only. Of these 143 were BRCA1/2 mutation carriers (mean age of onset 44 ± 9.97; range 28–79); 114 with breast cancer (including 14 with both breast and ovarian cancer); of whom 55 were diagnosed prior to age 42 years and 59 diagnosed after that age; and 29 with ovarian cancer only.

**Asymptomatic BRCA1/2 carriers** – 53 women (mean age 45.8 ± 11.24; range 27–79).

**Control** – 418 Ashkenazi individuals.

No statistically significant mean age differences were found between symptomatic and asymptomatic BRCA1/2 carriers and between these and non-BRCA1/2 carrier patients.

### Distribution of the I1307K APC mutation

Overall 57 (6%) I1307K APC mutation carriers were identified. The I1307K mutation was equally prevalent among patients with either sporadic (17/382; 4.6%) or inherited (10/143; 7%) breast and/or ovarian cancer (OR = 6.27, 95% CI = 0.7–57.7) and among

*© 2000 Cancer Research Campaign*

patients with breast cancer only (25/471; 5.3%) whether inherited (9/114; 7.9%) or sporadic (16/357; 4.9%) (OR = 1.6, 95% CI = 0.6–4.6).

The I1307K APC mutation was equally distributed among asymptomatic (7/53; 13.2%) and cancer manifesting BRCA1/2 carriers (10/143; 7%) (*P* = 0.14; OR = 2, 95% CI = 0.7–5.6). The I1307K APC mutation was equally distributed among BRCA1/2 carriers diagnosed with breast cancer prior to (4/55; 7.3%) and after (5/59; 8.5%) age 42 years (*P* = 0.545; OR = 0.8, 95% CI = 0.2–3.33). Among women with sporadic breast cancer the prevalence of the I1307K APC mutation was higher, although not statistically different, among those diagnosed after 42 years (15/276; 5.4%) than before (1/81; 1.2%) (*P* = 0.09; OR = 0.22, 95% CI = 0.03–1.7).

The overall distribution of I1307K APC mutation among BRCA1/2 carriers (17/196; 8.7%) was found to be significantly elevated compared to that observed among non-BRCA1/2 carriers (40/800; 5%) (*P* = 0.046; OR = 1.7, 95% CI = 1–3.1). The frequency of the I1307K allele was significantly higher among asymptomatic BRCA1/2 carriers (7/53; 13.2%) than among healthy controls (23/418; 5%) (*P* = 0.04; OR = 2.6, 95% CI = 1.1–6.4).

### DISCUSSION

Previous studies suggest that the APC I1307K missense mutation may act as a low penetrance gene/modifier both for sporadic breast cancer and breast cancer in BRCA1/2 mutation carriers (Redston et al, 1998; Woodage et al, 1998). In this study, we show that the I1307K polymorphism occurs at similar rates in the general Ashkenazi population and in women with sporadic breast (and/or ovarian) cancer. Yet among BRCA1/2 mutation carriers a higher frequency of I1307K mutation carriers was observed. The majority of patients with sporadic breast cancer patients who carried the I1307K APC mutation (15/16) had their breast cancer diagnosed after 42 years of age. No trend for 'early onset breast cancer' (< 42 years of age) was observed among double heterozygotes for both the APC I1307K polymorphism and a BRCA1/2 mutation. Taken together, these results do not support previously reported data indicating that this specific mutation, or rather polymorphism, confers a modest increased risk to developing breast cancer in Ashkenazi women (Redston et al, 1998; Woodage et al, 1998). Intriguingly, the rate of I1307K mutation in BRCA1/2 germline mutation carriers, was significantly higher than that observed in non-BRCA1/2 carriers with the asymptomatic BRCA1/2 mutation carriers having the highest rate of APC polymorphism. This observation seems to counteract the prediction (Redston et al, 1998; Woodage et al, 1998) that the effect of these two germline mutations would be additive, as anticipated from cancer susceptibility genes. A significantly higher rate of APC mutation carriers with sporadic breast cancer were indeed diagnosed after rather than before the age of 42 years, showing again that the APC polymorphism does not predispose to breast cancer.

Altogether, our results seem to weaken the notion that the I1307K APC gene polymorphism may act as a low penetrance gene/modifier in either sporadic or hereditary breast cancer. The high prevalence of APC mutation carriers among BRCA1/2 carriers does not seem to impinge upon morbidity even though some possibility of survivor bias should be taken into consideration. A plausible explanation is that the I1307K and BRCA1/2 mutated alleles are genetically connected because of Jewish ancestry and are not related with cancer risk.

### ACKNOWLEDGEMENTS

This work was performed in partial fulfillment of the requirements for the Ph.D. degree from the Sackler School of Medicine at the Tel-Aviv University for Yael Patael. This study was partially supported by the ICRF fund and the MECC to Dr Eitan Friedman.

### REFERENCES

Abeliovich D, Kaduri L, Lerer I, Weinberg N, Amir G, Sagi M, Zlotogora J, Heching N and Peretz T (1997) The founder mutations 185delAG and 5382insC in BRCA1 and 617delT in BRCA2 appear in 60% of ovarian cancer and 30% of early-onset breast cancer patients among Ashkenazi women. *Am J Hum Genet* **60**: 501–514

Bruchim Bar-Sade R, Kruglikova A, Modan B, Gak E, Hirsch-Yechezkel G, Theodor L, Novikov I, Gershoni-Baruch R, Risel S, Papa MZ, Ben-Baruch G and Friedman E (1998) The 185delAG BRCA-1 mutation originated before the dispersion of Jews in The Diaspora and not limited to Ashkenazim. *Hum Mol Genet* **7**: 801–806

Friedman LS, Szabo CI, Ostermeyer EA, Dowd P, Butler L, Park T, Lee MK, Goode EL, Sarah ER and King MC (1995) Novel inherited mutations and variable expressivity of BRCA1 alleles, including the founder mutation 185delAG in Ashkenazi Jewish families. *Am J Hum Genet* **57**: 1284–1297

Israel Cancer Registry (1992) Cancer in Israel: Ministry of Health, Jerusalem.

Kashiwaba M, Tamura G and Ishida MJ (1994) Aberrations of the APC gene in primary breast carcinoma. *J Cancer Res Clin Oncol* **120**: 727–731

Laken SJ, Petersen GM, Gruber SB, Oddoux C, Ostrer H, Giardiello FM, Hamilton SR, Hampel H, Markowitz A, Klimstra D, Jhanwar S, Winawer S, Offit K, Luce MC, Kinzler KW and Vogelstein B (1997) Familial colorectal cancer in Ashkenazim due to a hypermutable tract in APC. *Nature Genet* **17**: 79–83

Lerman LS, Fischer SG, Hurley I, Silverstein K and Lumelsky N (1984) Sequence-determined DNA separations. *Annu Rev Biophys Bioeng* **13P**: 399–423

Modan B, Gak E, Hirsch G, Bar-Sade Bruchim R, Theodor L, Lubin F, Ben-Baruch G, Beller U, Fishman A, Dgani R, Menczer J, Papa MZ and Friedman E (1996) High frequency of the 185delAG mutation in ovarian cancer in Israel. *JAMA* **76**: 1823–1225

Myers RM, Maniatis T and Lerman LS (1987) Detection and localization of single base changes by denaturing gradient gel electrophoresis. In *Methods Enzymology,* Wu R (ed.) pp 155, 501–527. Academic Press: San Diego

Neuhausen S, Gilewski T, Norton L, Tran T, McGuire P, Swensen J, Hampel H, Borgen P, Brown K, Skolnick M, Shattuck-Eidens D, Jhanwar S, Goldgar D and Offit K (1996) Recurrent BRCA2 617delT mutations in Ashkenazi Jewish women affected by breast cancer. *Nature Genet* **13**: 126–128

Patael Y, Figer A, Gershoni-Baruch R, Papa MZ, Rizel S, Chen R, Karasik A et al. (1999) A common origin of the I1307K APC polymorphism in Ashkenazi and non-Ashkenazi Jews. *EJHG* **7**: 555–559

Petrukhin L, Dangel J, Vanderveer L, Costalas J, Bellacosa A, Grana G, Dali and Godwin AK (1997) The I1307K APC mutation does not predispose to colorectal cancer in Jewish Ashkenazi breast and breast–ovarian cancer kindreds. *Cancer Res* **57**: 5480–5484

Redston M, Nathanson KL, Yuan ZQ, Neuhausen SL, Satagopan J, Wong N, Yang D, Nafa D, Abrahamson J, Ozcellik H, Antin-Ozerkis D, Andrulis I, Daly M, Pinski L, Shrag D, Galinger S, Kaback M, King MC, Woodage T, Brody LC, Godwin A, Warner E, Weber B, Foulkes W and Offit K (1998) The APC I1307K allele and breast cancer risk. *Nature Genet* **20**: 13–14

Rohlfs EM, Learning WG, Friedman KJ, Couch FJ, Weber BL and Silverman LM (1997) Direct detection of mutations in the breast and ovarian cancer susceptibility gene BRCA1 by PCR-mediated site-directed mutagenesis. *Clin Chem* **43**: 24–29

Thompson AM, Morris RG, Wallase M, Willie AH, Steel CM and Carter DC (1993) Allele loss from 5q21 (APC-MCC) and 18q21 (DCC) and DCC mRNA expression in breast cancer. *Br J Cancer* **68**: 64–68

Tonin P, Weber B, Offit K, Couch F, Rebbeck TR, Neuhausen S, Godwin AK, Daly M, Wagner-Costalos J, Berman D, Grana J, Fox E, Kane MF, Kolodner RD, Krainer M, Haber DA, Struewing JP, Warner E, Rosen B, Lerman C, Peshkin B, Norton L, Serova O, Foulkes WD and Graber JE (1996) Frequency of recurrent BRCA1 and BRCA2 mutations in Ashkenazi Jewish breast cancer families. *Nature Med* **2**: 1179–1183

Woodage T, King SM, Wacholder S, Hartge P, Struewing JP, McAdams M, Laken SJ, Tucker MA and Brody LC (1998) The *APC* I1307K allele and cancer risk in a community-based study of Ashkenazi Jews. *Nature Genet* **20**: 62–65

© 2000 Cancer Research Campaign

## References

[PDF_00241] Abeliovich D., Kaduri L., Lerer I., Weinberg N., Amir G., Sagi M., Zlotogora J., Heching N., Peretz T. (1997). The founder mutations 185delAG and 5382insC in BRCA1 and 6174delT in BRCA2 appear in 60% of ovarian cancer and 30% of early-onset breast cancer patients among Ashkenazi women.. Am J Hum Genet.

[PDF_00246] Bar-Sade R. B., Kruglikova A., Modan B., Gak E., Hirsh-Yechezkel G., Theodor L., Novikov I., Gershoni-Baruch R., Risel S., Papa M. Z. (1998). The 185delAG BRCA1 mutation originated before the dispersion of Jews in the diaspora and is not limited to Ashkenazim.. Hum Mol Genet.

[PDF_00251] Friedman L. S., Szabo C. I., Ostermeyer E. A., Dowd P., Butler L., Park T., Lee M. K., Goode E. L., Rowell S. E., King M. C. (1995). Novel inherited mutations and variable expressivity of BRCA1 alleles, including the founder mutation 185delAG in Ashkenazi Jewish families.. Am J Hum Genet.

[PDF_00256] Kashiwaba M., Tamura G., Ishida M. (1994). Aberrations of the APC gene in primary breast carcinoma.. J Cancer Res Clin Oncol.

[PDF_00258] Laken S. J., Petersen G. M., Gruber S. B., Oddoux C., Ostrer H., Giardiello F. M., Hamilton S. R., Hampel H., Markowitz A., Klimstra D. (1997). Familial colorectal cancer in Ashkenazim due to a hypermutable tract in APC.. Nat Genet.

[PDF_00262] Lerman L. S., Fischer S. G., Hurley I., Silverstein K., Lumelsky N. (1984). Sequence-determined DNA separations.. Annu Rev Biophys Bioeng.

[PDF_00265] Modan B., Gak E., Sade-Bruchim R. B., Hirsh-Yechezkel G., Theodor L., Lubin F., Ben-Baruch G., Beller U., Fishman A., Dgani R. (1996). High frequency of BRCA1 185delAG mutation in ovarian cancer in Israel. National Israel Study of Ovarian Cancer.. JAMA.

[PDF_00269] Myers R. M., Maniatis T., Lerman L. S. (1987). Detection and localization of single base changes by denaturing gradient gel electrophoresis.. Methods Enzymol.

[PDF_00272] Neuhausen S., Gilewski T., Norton L., Tran T., McGuire P., Swensen J., Hampel H., Borgen P., Brown K., Skolnick M. (1996). Recurrent BRCA2 6174delT mutations in Ashkenazi Jewish women affected by breast cancer.. Nat Genet.

[PDF_00276] Patael Y., Figer A., Gershoni-Baruch R., Papa M. Z., Risel S., Shtoyerman-Chen R., Karasik A., Theodor L., Friedman E. (1999). Common origin of the I1307K APC polymorphism in Ashkenazi and non-Ashkenazi Jews.. Eur J Hum Genet.

[PDF_00280] Petrukhin L., Dangel J., Vanderveer L., Costalas J., Bellacosa A., Grana G., Daly M., Godwin A. K. (1997). The I1307K APC mutation does not predispose to colorectal cancer in Jewish Ashkenazi breast and breast-ovarian cancer kindreds.. Cancer Res.

[PDF_00283] Redston M., Nathanson K. L., Yuan Z. Q., Neuhausen S. L., Satagopan J., Wong N., Yang D., Nafa D., Abrahamson J., Ozcelik H. (1998). The APCI1307K allele and breast cancer risk.. Nat Genet.

[PDF_00288] Rohlfs E. M., Learning W. G., Friedman K. J., Couch F. J., Weber B. L., Silverman L. M. (1997). Direct detection of mutations in the breast and ovarian cancer susceptibility gene BRCA1 by PCR-mediated site-directed mutagenesis.. Clin Chem.

[PDF_00292] Thompson A. M., Morris R. G., Wallace M., Wyllie A. H., Steel C. M., Carter D. C. (1993). Allele loss from 5q21 (APC/MCC) and 18q21 (DCC) and DCC mRNA expression in breast cancer.. Br J Cancer.

[PDF_00295] Tonin P., Weber B., Offit K., Couch F., Rebbeck T. R., Neuhausen S., Godwin A. K., Daly M., Wagner-Costalos J., Berman D. (1996). Frequency of recurrent BRCA1 and BRCA2 mutations in Ashkenazi Jewish breast cancer families.. Nat Med.

[PDF_00301] Woodage T., King S. M., Wacholder S., Hartge P., Struewing J. P., McAdams M., Laken S. J., Tucker M. A., Brody L. C. (1998). The APCI1307K allele and cancer risk in a community-based study of Ashkenazi Jews.. Nat Genet.

